# Supplementary material for: Resource selection of a montane endemic: Sex-specific differences in white-bellied voles (Microtus longicaudus leucophaeus)
Source: PLoS One. 2020 Nov 9;15(11):e0242104. doi: 10.1371/journal.pone.0242104 (PMC7652259; doi:10.1371/journal.pone.0242104)
Supplement: S1 Table — (DOCX) [file pone.0242104.s001.docx]

**Supporting information**

**S1 Table. Patch scale *a priori* 4^th^ order conditional mixed-effects logistic regression models.**

| **Group** | **Covariates** | **AIC** | **∆AIC** |
| --- | --- | --- | --- |
| **All voles** | Understory cover + Canopy cover + Bare ground + Grass + Forb + Log + Coarse woody debris + Distance to water + Distance to road | 496.0 | 0.0 |
|  | Understory cover + Canopy cover + Grass + Log + Distance to water | 537.6 | 41.6 |
|  | Understory cover + Grass + Distance to water | 552.3 | 56.3 |
|  | Grass + Forb + Log + Coarse woody debris | 554.5 | 58.5 |
|  | Understory cover + Canopy cover | 561.2 | 65.2 |
| **Females** | Understory cover + Canopy cover + Bare ground + Grass + Forb + Log + Coarse woody debris + Distance to water + Distance to road | 327.8 | 0.0 |
|  | Understory cover + Canopy cover + Grass + Log + Distance to water | 350.4 | 22.6 |
|  | Understory cover + Grass + Distance to water | 360.9 | 33.1 |
|  | Understory cover + Canopy cover | 362.4 | 34.6 |
|  | Grass + Forb + Log + Coarse woody debris | 370.3 | 42.5 |
| **Males** | Understory cover + Canopy cover + Bare ground + Grass + Forb + Log + Coarse woody debris + Distance to water + Distance to road | 160.7 | 0.0 |
|  | Grass + Forb + Log + Coarse woody debris | 170.9 | 10.2 |
|  | Understory cover + Canopy cover + Grass + Log + Distance to water | 189.8 | 29.2 |
|  | Understory cover + Grass + Distance to water | 193.2 | 32.6 |
|  | Understory cover + Canopy cover | 202.1 | 41.4 |

AIC, Akaike information criterion.

ΔAIC is the difference in AIC values between each model and the lowest AIC model.
